# Prevalence of anxiety and depression in high school students of Karachi, Pakistan

**DOI:** 10.12669/pjms.38.4.5093

**Published:** 2022

**Authors:** Saima Ibbad, Lubna Ansari Baig, Zaeema Ahmer, Farhana Shahid

**Affiliations:** 1 Dr. Saima Ibbad, MBBS, MSPH. Lecturer, APPNA Institute of Public Health, Jinnah Sindh Medical University, Rafiqui H.J Road, Karachi 75510, Pakistan; 2 Prof. Dr. Lubna Ansari Baig, MBBS, FCPS, MPH, PhD. Founding Dean and Chairperson, APPNA Institute of Public Health, Jinnah Sindh Medical University, Rafiqui H.J Road, Karachi 75510, Pakistan; 3 Dr. Zaeema Ahmer, MBBS, MSPH. Assistant Professor, APPNA Institute of Public Health, Jinnah Sindh Medical University, Rafiqui H.J Road, Karachi 75510, Pakistan; 4 Dr. Farhana Shahid, MBBS, MPH, PhD. Associate Professor and Head of Department, Public Health, Faculty of Life Sciences SZABIST, Karachi, Pakistan

**Keywords:** Anxiety, Depression, High school students, Karachi

## Abstract

**Objectives:**

Anxiety and depression are commonly occurring mental disorders in school-going students and if not considered can result in a worse outcome. The objective of our study was to determine the prevalence, risk factors and relationship of anxiety and depression with different variables among high school students of Karachi.

**Methods:**

This cross-sectional study was conducted in October - November 2020 in government and private high schools of four districts of Karachi. Two stage cluster sampling was used to select study sites with 400 students inducted. Data was collected by using Aga Khan University Scale of anxiety and depression. Relationship of outcome with predictor variables was examined by applying univariate and multivariate logistic regression analysis.

**Results:**

Overall 53.2% participants reported anxiety and depression out of which 78.8% were females. Participants who were studying in private schools (OR 0.39, CI 0.21-0.69 at 95%, p=0.002) and had mothers who were housewives (OR 0.28, CI 0.09-0.83 at 95%, p=0.022) were less likely to develop anxiety and depression. Participants whose fathers and mothers had a history of anxiety and depression were three times more likely to develop anxiety and depression (OR 3.12, CI 1.52-6.41 at 95%, p=0.002) and (OR 3.02, CI 1.39-6.59 at 95%, p=0.005) respectively.

**Conclusion:**

The study found a high prevalence of anxiety and depression among high school students. Female students of public sector school and those who had a family history of anxiety and depression were more likely to develop it. Early detection and management of anxiety and depression by screening are necessary to overcome this burden.

## INTRODUCTION

Anxiety and depression are serious mental health problems, causing high morbidity around the world. Adolescent age, when student joins high school is crucial for an individual’s mental development and as such efforts must be focused on solving problems like stress, anxiety, and depression.^1^ Treating such conditions is not an easy task considering the multifaceted physical, mental and biological changes that an individual goes through during this period.^2^ Mental issues during this age are a major burden on the health system and particularly because of their importance in an individual’s growth. If left untreated, psychiatric disorders might have an impact on the overall life of an individual ranging from poor grades in school to abuse of substances, and in extreme cases, even suicide becomes a possibility.^3^

According to World Health Organization (WHO), the results of several studies show that the occurrence rate with regards to psychiatric disorders is around 22% in various settings.^4^,^5^ A report published in 2015 on the global burden of depression and other mental disorders reported 322 million people as suffering from depression over the world. This had increased by 18.4% from 2005 to 2015. The prevalence varies among different ages with 8.7% prevalence globally in children aged 15-19 years.^6^ One of the studies conducted amongst boys from a secondary school in Saudi Arabia revealed 41% of the students suffering from depression, half of them reported to have anxiety and stress was found in approximately 35% of the boys.^7^ A different study conducted in a secondary girl’s school in Abha, Saudi Arabia showed 42% of the girls suffering from depression with 68% of the total population experiencing anxiety while around half of the girls suffering from stress.^8^ A study on emotional and behavioral problems faced by school going children of Karachi reported higher prevalence as compared to other countries.^9^ In another recent survey in Rawalpindi, one in four adolescents in public schools of rural Rawalpindi were identified as psychosocially distressed.^10^

Several risk factors of anxiety and depression has been identified from previous literature which include family and personal history of depression, academic difficulties, economic instability, diagnosis of a serious illness, death of a loved one, separation of parents, alcohol consumption, planning and / or attempt to suicide.^11^,^12^ A study also reported that depression could be related to academic difficulties resulting in the loss of interest in daily activities.^13^

Pakistan’s rapidly growing population reflects the need to build an evidence-based policy for developing effective strategies to tackle the menace of anxiety and depression and its related disorders.^14^ Not enough data and information is available to understand the magnitude of this problem in our young population. Hence our objective was to estimate the prevalence and assess the risk factors associated with anxiety and depression among high school students in Karachi, Pakistan.

## METHODS

This cross-sectional study and was carried out in October - November 2020 in government and private high schools of all four districts of Karachi after obtaining ethical approval from Institutional Review Board of Jinnah Sindh Medical University (JSMU/IRB/2020/330). Two staged cluster sampling technique was used to select schools and study participants. At first stage, a list of all schools and colleges providing higher secondary school education in Karachi was obtained from Sindh Education Department. In the second stage, ten schools were selected from each district and approached for data collection. There were some schools from District South and District West which refused permission for the study due to COVID and privacy reasons. Those schools were dropped whereas schools that granted permission were followed up. Hence three-four schools from each district were finalized for data collection. Sample size was calculated from OpenEpi calculator, with previous prevalence of 57.65% in school going adolescents.^15^ Taking 5% precision and 95% confidence level, sample size came out to be 376. However, it was inflated to 400 to account for missing data with 100 study participants recruited from each district. All students of the selected schools were eligible to participate in the study. There were some students who refused to participate in the study citing reasons of discomfort and fear of parent’s disapproval. They were then excluded from the study. Students were recruited by applying non-probability convenience sampling technique due to COVID restrictions.

Data was collected on age, gender, type of school, family history of anxiety and depression disorder, parents’ profession, other physical disability and educational history. Aga Khan University-Anxiety and Depression Scale (AKU-ADS) questionnaire was used to assess the prevalence of anxiety and depression among high school students after obtaining formal permission from authors.^16^ This is a 24-item, 5-point Likert Scale (always, mostly, sometimes, never, don’t know responses) validated tool with a score of more than 19 indicating some form of anxiety and depression. The anxiety and depression scale was found reliable in our respondents. The scale used in the study showed good reliability with Cronbach’s Alpha of 0.824.

The selected schools were approached by the principal investigator and formal permission of relevant authorities of school was taken. Consent forms were handed over to the principal to take the permission from the parents of those students who were below the age of 18 years while written consent was taken from students aged 18 and above. In both the cases, participants were also informed about right to refuse or withdraw at any stage of study. They were assured that their privacy would be respected and responses received would be kept confidential. The filled forms were assessed to check whether the questionnaire has been filled completely and correctly. At the end of the study, students, parents and principals of schools were also provided with a list of psychologists and psychiatrists working in government and private sector of Karachi for referral upon diagnosis of anxiety and depression.

Data was entered in Statistical Package for Social Science (SPSS) version 20. After cleaning, data was analyzed in the same package. Frequencies, distribution and percentages were calculated for categorical variables. To determine the relationship between factors and outcome, Odds Ratio with 95% confidence level was calculated using univariate and multivariate logistic regression analysis. A p-value of less than 0.05 was considered statistically significant.

## RESULTS

The total sample size was 400 students with mean age of study participants reported as 17.23 ± 1.11. Majority, 61.5% of the participants belonged to the age group 15-17 years with 72% females. There were 61.2% of the participants from private schools with 57% of the students reporting good academic grades and 91.7% of them never failing in previous class. There was no physical disability in 99% study participants and 91.5% didn’t report any history of recent trauma. There were 15.5% of students who reported a positive history of anxiety and depression in their fathers whereas 19% of students reported a positive history of anxiety and depression in their mothers (Table-I).

**Table-I:** Background Information of Study Participants (n=400).

Variable	Mean ± SD
Mean Age of Study Participants	17.2 ± 1.1
	%(n)
***Age in Categories***	
15-17 Years	61.5 (246)
18-19 Years	38.5 (154)
***Gender***	
Male	28 (112)
Female	72 (288)
***Type of School***	
Government	38.8 (155)
Private	61.2 (245)
***Grading in Studies***	
Excellent	22 (88)
Good	57 (228)
Satisfactory	17 (68)
Unsatisfactory	1.8 (7)
Don’t Know	2.3 (9)
***Ever Failed in Previous Class***	
Never	91.7 (367)
Yes	8.3 (33)
***Physical Disability***	
No	99 (396)
Yes	1 (4)
***History of Recent Trauma***	
No	91.5 (366)
Yes	8.5 (34)
***Father History Of Depression/Anxiety***	
No	84.5 (338)
Yes	15.5 (62)
***Mother History of Depression/Anxiety***	
No	81 (324)
Yes	19 (76)

**Fig.1:**
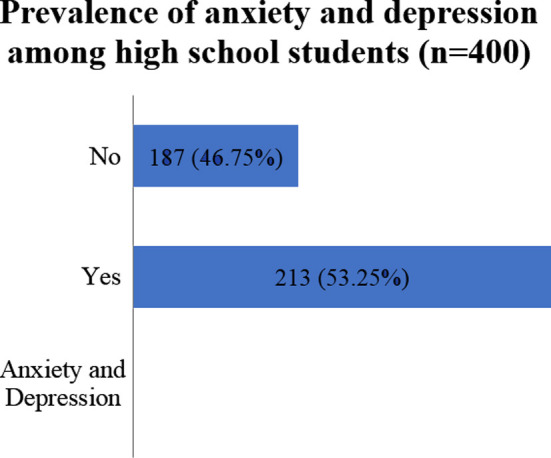
Prevalence of anxiety and depression among high school students (n=400).

The prevalence of anxiety and depression among high school students was 53.25% (Fig.1).

Association of anxiety and depression with different variables of the study participants showed that females (OR 2.05, CI 1.18 – 3.56, p-value = 0.010) were twice more likely to develop anxiety and depression as compared to males. Students whose fathers (OR 3.02, CI 1.39 – 6.59. p- value = 0.005) and mothers (OR 3.12, CI 1.52 – 6.41, p-value = 0.002) were suffering from anxiety and depression were thrice more likely to have develop it as compared to students whose fathers and mothers were not having anxiety and depression respectively. Students of private schools (OR 0.39, CI 0.21- 0.69, p-value = 0.002) and whose mothers were housewives (OR 0.28, CI 0.09 – 0.83, p-value = 0.022) were less likely to develop anxiety and depression as compared to others (Table-II).

**Table-II:** Regression Analysis of Anxiety and Depression with Sociodemographic Variables of the Study Participants (n=400).

Study Variable	Unadjusted OR (95% CI)	p-value	Adjusted OR (95% CI)	p-value
***Age Categories***				
15-17 Years	*Reference*		*Reference*	
18-19 Years	1.13(0.75 – 1.70)	0.537	0.72(0.44 – 1.20)	0.219
***Gender***
Male	*Reference*		*Reference*	
Female	2.08(1.33 – 3.25)	0.001	2.05(1.18 – 3.56)	0.010
***Type of School***				
Government	*Reference*		*Reference*	
Private	0.56(0.37 – 0.84)	0.006	0.39(0.21 – 0.69)	0.002
***Father Profession***				
Govt. Employee	*Reference*		*Reference*	
Pvt. Employee	1.07(0.61 – 1.87)	0.804	0.88(0.44 – 1.74)	0.727
Businessman	1.53(0.83 – 2.82)	0.169	1.66(0.78 – 3.54)	0.186
Unemployed	1.03(0.36 – 2.93)	0.954	0.84(0.23 – 3.01)	0.796
***Mother Profession***				
Employed	*Reference*		*Reference*	
House Wife	0.27(0.10 – 0.68)	0.006	0.28(0.09 – 0.83)	0.022
***Student Grading***				
Excellent	*Reference*		*Reference*	
Good	1.29(0.79 – 2.12)	0.302	1.07(0.60 – 1.90)	0.811
Satisfactory	1.54(0.81 – 2.91)	0.183	2.10(0.96 – 4.61)	0.063
Unsatisfactory	6.87(0.79 – 59.52)	0.080	5.00(0.38 – 64.96)	0.219
Don’t Know	2.29(0.53 – 9.75)	0.261	1.82(0.31 –10.56)	0.504
***Father Suffered from Anxiety/Depression***				
No	*Reference*		*Reference*	
Yes	3.99(2.09 – 7.64)	0.000	3.02(1.39 – 6.59)	0.005
***Mother Suffered from Anxiety/Depression***				
No	*Reference*		*Reference*	
Yes	3.51(1.98 – 6.22)	0.000	3.12(1.52 – 6.41)	0.002

## DISCUSSION

This is perhaps the first ever study conducted to assess prevalence of anxiety and depression among high school students in four districts of Karachi in Pakistan. The results of this study show that prevalence of anxiety and depression in high school students was quite high. This finding is similar to another study that was carried out in Haryana, India, that found similar prevalence of depression among school going adolescents.^17^ Due to similar cultural contexts, this finding can be explained.

Our study also found a high prevalence of anxiety and depression among our female participants. The finding of this study is consistent with numerous other previous studies conducted on same populations with higher prevalence reported among female students. A study conducted at Al-Qassim region in Saudi Arabia found similar prevalence of anxiety and depression in female students of high schools.^2^ Another study carried out in India also found higher prevalence of depression, anxiety and stress in females.^15^ A similar study conducted in India on prevalence and correlates of depression among school children in Amritsar reported high levels of depression in female students.^18^ Another study on prevalence of depression in higher secondary students at Bhopal, India also reported female students were more prone to depression.^19^ A study conducted in Turkey on prevalence of depression in higher education students also showed higher prevalence of depression in female participants.^3^ Females have been found to be depressed and anxious in their professional colleges as well as cited by numerous local studies.^20^-^22^ These findings related to higher prevalence in females can be explained by findings of a previous study which stated that the sexual abuse and social limitations like gathering with companions could be reason behind higher prevalence in female; however current study didn’t assess these factors so they can’t be excluded.^23^

A study was conducted in Iran on females who were studying in secondary school to assess level of depression by using Persian version of depression scale.^1^ Study reported significant difference in levels of depression in participants on basis of type of school, students who were enrolled in public schools showed higher prevalence as compared to those enrolled in private school. This finding relates with current study where majority of participants reported anxiety and depression belonged to government schools. This could be because students of private school are assumed to belong to a higher socioeconomic stratum and are more likely to engage in social gatherings with companions and get greater motivation to continue their education without anxiety and depression. Moreover, comfortable environment and facilities, modern teaching methodologies with updated curriculum in private schools could be reason of less prevalence of anxiety and depression among this group.^24^

Iranian study also revealed that mother of participants reported any level of depression were housewives, however there was no significant difference between employed or unemployed status of mothers whereas our study found significant difference in this category.^1^ In our study, participants whose mothers were housewives were less likely to develop anxiety and depression. This could be attributed to the constant presence and nurturing of stay-at-home mothers.^23^ Our study contradicts the study carried out in Bhopal India on students of 9th and 10th classes where depression in participants was significantly associated with mothers who were housewives.^19^ In this study, factors like living in joint family were also explored which we did not do, which could be a possible reason for this observation.

Research conducted in Iran showed significant correlation of family history of psychiatric disorder with prevalence of depression in their children.^23^ This finding was same as our study, where there was significant association of family history of depression in either parent with prevalence of depression in participants after adjustment with other confounders. This finding can be justified by available literature of clinical psychology which reports that family history of depression is a predictor or risk factor of depression.^23^

### Limitations of the study:

This include cross-sectional nature of study hence temporal relationship could not be established; plus the study selected schools on the basis of convenience due to lack of sufficient resources and time limitation. The other limitation was that the study used self-administered data collection methods which could have caused participant’s bias.

## CONCLUSION

The study reported high prevalence of anxiety and depression among adolescents. The study found that being a female student of public sector school and those who had a family history of depression were more likely to develop anxiety and depression. We recommend placing a certified counselor in high schools for proper guidance of students of this age group. Additionally those students who report with anxiety and depression symptoms must be referred for proper diagnosis and treatment with information readily available on local resources for help if needed.

### Author’s Contribution:

**SI:** Proposed the study design, conceptualized the project and collected data.

**LB:** Supervised and critically analyzed the project throughout its duration.

**ZA:** Wrote the first draft of the manuscript.

**FS:** Analyzed the data and revised the manuscript critically for important intellectual content.

All authors checked and approved the final version of the manuscript and are responsible for the integrity of the work.
